# Delaying Screening Until Covered? Changes in Lung Cancer Screening at the Age of Nearly‐Universal Medicare Insurance

**DOI:** 10.1111/1475-6773.14638

**Published:** 2025-05-08

**Authors:** Marcelo C. Perraillon, Adam Warren, Lenka Goldman, Jamie L. Studts, Rebecca M. Myerson

**Affiliations:** ^1^ Department of Health, Systems, Management and Policy University of Colorado Anschutz Medical Campus Aurora Colorado USA; ^2^ University of Colorado Anschutz Medical Campus Aurora Colorado USA; ^3^ American College of Radiology Reston Virginia USA; ^4^ Division of Medical Oncology, Department of Medicine University of Colorado Anschutz Medical Campus Aurora Colorado USA; ^5^ Department of Population Health Sciences University of Wisconsin School of Medicine and Public Health Madison Wisconsin USA

**Keywords:** health insurance, lung cancer, lung cancer screening, Medicare, shared decision‐making

## Abstract

**Objective:**

To estimate changes in lung cancer screening at age 65, the age of nearly universal Medicare coverage.

**Study Setting and Design:**

Screening reduces lung cancer mortality but is underutilized. We used a regression discontinuity design to measure the impact of nearly universal Medicare coverage at age 65 on first‐time receipt of screening (primary outcome) and the proportion of screened individuals with detected lung cancer (secondary outcome).

**Data Sources and Analytic Sample:**

First‐time screens at age 60–69 in the American College of Radiology's Lung Cancer Screening Registry data, 2015–2020.

**Principal Findings:**

Nearly‐universal access to Medicare at 65 increased first‐time lung cancer screening by 5450 per year (CI 4911–5990), a 41% increase compared to age 64. Eighty‐nine percent of additional screens were among people who met screening eligibility criteria. Increases at age 65 were larger in rural areas than nonrural areas (52% vs. 39%) and were similar for men and women (41% and 42%). There was no statistically significant change in the proportion of screened individuals with lung cancer detected.

**Conclusion:**

First‐time receipt of lung cancer screening increases at age 65, particularly among people in rural areas. Cancer detection rates did not worsen, suggesting screening remained well targeted as it increased.

**Summary**

- What is known on this topic
  ○ Screening with low‐density computed tomography (LDCT) among high‐risk individuals substantially reduces lung cancer mortality, the leading cause of cancer mortality in the United States.
  ○ For those who qualify for LDCT and are insured, screening is usually exempt from cost‐sharing, but confirmatory procedures and treatment are not.
  ○ LDCT screening is underutilized among high‐risk individuals; barriers include lack of awareness about screening benefits, stigma, inadequate access to healthcare, and concerns about downstream treatment costs.
- What this study adds
  ○ Using data from the national lung cancer screening registry, we found a large increase in first‐time lung cancer screens at age 65, the age of nearly universal Medicare coverage.
  ○ The increase in screening was similar for men and women and larger in rural areas, where baseline screening rates are lower.
  ○ Lung cancer detection rates did not change at age 65, suggesting screening remained well‐targeted despite its increase after gaining Medicare.

## 1 Introduction

Lung cancer is the leading cause of cancer mortality in the United States [1]. Among adults at high risk of lung cancer based on their smoking history and age, clinical trials found that annual screening with low‐dose computed tomography (LDCT) reduced the risk of lung cancer mortality by one‐fifth relative to screening with chest radiographs [2]. Since 2013, the US Preventive Services Task Force (USPSTF) has recommended annual lung cancer screening via LDCT for high‐risk individuals [3]. Despite the benefits of screening, the procedure remains vastly underutilized. Less than 20% of eligible individuals received screening in 2022, with large variability across states [4, 5, 6, 7, 8, 9]. More recent guidelines from USPSTF, the Centers for Medicare and Medicaid Services, and the American Cancer Society expanded the group of adults who should receive lung cancer screening, underscoring the need for increasing uptake among eligible adults [10, 11, 12].

Several provider and patient factors may explain low rates of screening uptake. Patient preferences, perception of stigma, barriers to health insurance coverage, a lack of knowledge about free screening for qualified individuals, provider unawareness of eligibility criteria, and difficulty identifying eligible patients are among potential reasons [13, 14, 15]. For those who do not have insurance, the annual cost of lung cancer screening can be high. Researchers who called screening centers found that out‐of‐pocket costs of screening ranged from $49 to $2409 in 2019 (average: $583) [16]. A cost‐effectiveness study based on the National Lung Screening Trial used a price of $284.9 for LDCT in 2014 [17], and an online cost transparency tool reported an estimated national price of $449 in 2025 [18]. While health insurance can substantially reduce the cost to patients, those who do not meet the USPSTF eligibility or receive screens out‐of‐network, when an in‐network provider is available, may still incur high cost‐sharing given the large proportion of commercial health plans with deductibles [15, 19, 20, 21].

Gaining Medicare coverage at 65 might increase lung cancer screening initiation for several reasons [22]. First, acquiring health insurance reduces the cost of screening for previously uninsured individuals [23, 24, 25]. Second, the addition of Medicare coverage provides additional protection from cost‐sharing for the already insured. While the USPSTF “B” rating LDCT screening ensures that health insurance plans cover screening without cost‐sharing for eligible adults, subsequent tests, procedures, and treatment are not exempt from cost‐sharing [17, 26]. Third, Medicare provides preventive services with no cost‐sharing (Welcome to Medicare Visit and Annual Wellness Visit), which increase screening rates [27, 28, 29, 30, 31]. A prior study using survey data found an increase of 16.2 percentage points in lung cancer screening among men at age 65, with no significant change for women [32]. However, this study used a small sample that was not nationally representative and used self‐reported data on lung cancer screening that could be subject to recall bias.

Despite the benefits of screening for people at high risk, concerns exist about the costs and harms associated with overscreening and overdiagnosis [33]. Thus, it is relevant to patients and policymakers to establish whether lung cancer screening remains targeted to high‐risk people as screening rates rise (e.g., whether lung cancer detection rates remain high and whether the screened population meets USPSTF criteria for recommended screening based on their age and smoking history) [6, 34]. To encourage screening among people at high risk, Medicare requires that the first screens be preceded by a shared decision‐making conversation about the potential benefits and harms of screening given their personal risk factors [35]. Previous studies did not capture the lung cancer detection rates of the screened population and lacked national estimates of the pack‐year history of screened people, preventing clear conclusions about how Medicare coverage affects targeting of screening to people at high risk [32]. Given the high mortality associated with late‐stage lung cancer, delaying screening until 65 may increase mortality for people with high lung cancer risk.

The goal of this study was to assess the impact of nearly‐universal access to Medicare at age 65 on the initiation of lung cancer screening and detection of lung cancer. We used a regression discontinuity approach and data from a national registry to compare lung cancer screening and detection outcomes for people just under versus just over age 65.

## 2 Methods

### 2.1 Data

We used 2015–2020 data from the American College of Radiology's Lung Cancer Screening Registry, a nationwide registry of LDCT lung cancer screening. During the study period, facilities accepting Medicare were required to submit data on screened patients, including patients without Medicare insurance [7]. These data include smoking history (current or former, and number of pack‐years of smoking history); rurality of the facility location; reported use of shared decision‐making; and whether lung cancer was detected (these fields are required to be reported for all screened patients) [36]. Despite the high rate of missingness, we included race, ethnicity, and education as reported by the registry to better describe the study sample.

To capture the initiation of screening, we limited the data to the first reported screen for each person. The dataset included over 2.4 million initial lung cancer screens for people of any age. The primary outcome of interest was the number of people receiving lung cancer screening. The secondary outcome was lung cancer detection among those screened.

### 2.2 Statistical Analysis

We employed a regression discontinuity design (RDD) to determine the causal relationship between gaining Medicare coverage and lung cancer screening [32, 37]. The internal validity of this method relies in part on the arbitrariness of age 65 as the threshold for Medicare eligibility relative to receipt of lung cancer screening and on individuals unable to precisely manipulate age‐based eligibility. Because cancer screening tends to increase with age, the estimation requires that regression models correctly specify the relationship between age and receipt of screening, a potential confounder [38]. RDD can only identify causal effects at age 65, which in this study is the effect of interest since our goal is to estimate the impact of the onset of nearly‐universal Medicare coverage at 65. Because of the large sample size, we used a narrow bandwidth of 5 years around 65 to strengthen internal validity.

We estimated negative binomial models grouped by age and year to account for the overdispersion of screen counts. The dependent variable was the number of first‐time screens. We measured counts (number of people screened) rather than rates (proportion of eligible people screened). Registry data do not include the size of the population eligible for screening in the facility's catchment area, and the identities of facilities were not disclosed, so it was not possible to use external data to calculate a denominator. The main predictor of interest was an indicator variable for age over 65. To account for trends in screening by age, the models included a quadratic polynomial in age (centered at 65). Interaction terms were used to allow trends by age to differ above versus below age 65. Models included indicator variables for the year of the screen. We used regression weights with a triangular kernel to upweight observations closer to age 65. Individual‐level logistic regression models with similar parametric structure were used to estimate changes in the probability of lung cancer detection at age 65. We report marginal effects.

To assess the sensitivity of the findings to model specification, we conducted alternate analyses that used different windows of data around age 65 (a larger window of 6 years and a smaller window of 4 years); used linear trends, rather than a quadratic polynomial, to adjust for age; and omitted weights. We also conducted stratified analyses based on lung cancer risk, smoking history, and demographic factors (male or female sex, and rural vs. non‐rural facility location). High lung cancer risk was defined as meeting the USPSTF criteria for lung cancer screening eligibility in place at the time (adults aged 55 or older who have at least a 30‐pack‐year smoking history and currently smoke or quit within the past 15 years) [3]. In additional analyses, we separately studied people meeting USPSTF criteria who had 30–39 pack‐years of smoking history versus people with 40 or more pack‐years of smoking history.

Analyses were conducted using the R statistical software. We assessed statistical significance at the *p* < 0.05 level using two‐sided tests. The Institutional Review Board approved the research.

## 3 Results

The analytical sample included 877,915 initial lung cancer screens for individuals aged 60–69. Women accounted for 47% of the sample, and 59% of screened individuals were current smokers. Most screenings were conducted in metropolitan or suburban areas, with 19% in rural areas. Data on race, ethnicity, and education was missing for many individuals. Table 1 presents the characteristics of screened individuals in two age groups, 60–64 and 65–69. Individuals who were screened at 60–64 and those screened at 65–69 had similar characteristics except for smoking status (62% vs. 56%, respectively). Most people received shared decision‐making regardless of whether they were screened above or below age 65, and 89% of those screened met eligibility criteria.

**TABLE 1:** Characteristics of people whose first lung cancer screen occurred before versus after age 65.

	Age 60–64 (*N* = 427,139)		Age 65–69 (*N* = 450,776)	
	*N*	%	*N*	%
Gender				
Female	205,016	48%	211,166	47%
Male	220,866	52%	238,422	53%
Other, unknown, missing	1257	0.3%	1188	0.3%
Ethnicity				
Hispanic or Latino	3693	0.9%	3521	0.8%
Not Hispanic or Latino	149,770	35%	158,499	35%
Missing	273,676	64%	288,756	64%
Race				
Asian	1739	0.4%	1838	0.4%
Black	16,605	4%	15,770	4%
Multirace	30,045	7%	31,720	7%
Native American, Alaskan Native, or Pacific Islander	1191	0.3%	1205	0.3%
White	178,809	42%	189,719	42%
Missing	198,750	47%	210,524	47%
Education				
High school	12,769	3%	12,310	2.7%
Less than high school	3809	0.9%	3516	0.8%
Post‐high school degree completed	381,639	89%	406,380	90%
Missing	28,922	7%	28,570	6%
Smoking status				
Currently smoke	266,490	62%	252,063	56%
Formerly smoked	153,615	36%	191,501	42%
Never smoked	775	0.2%	864	0.2%
Ever‐smoked, current status Unknown	3065	0.7%	3094	0.7%
Missing	3194	0.8%	3254	0.7%
Location				
Metropolitan	184,460	43%	195,225	43%
Rural	79,103	19%	84,110	19%
Suburban	163,576	38%	171,441	38%
Shared decision‐making received				
	316,123	74%	2822	75%

*Note:* The null hypothesis of no difference in the distribution of baseline characteristics is rejected for all baseline characteristics using chi‐squared tests at a 5% significance level (two‐tailed). Data from the American College of Radiology's Lung Cancer Screening Registry (2015–2020).

**FIGURE 1:**
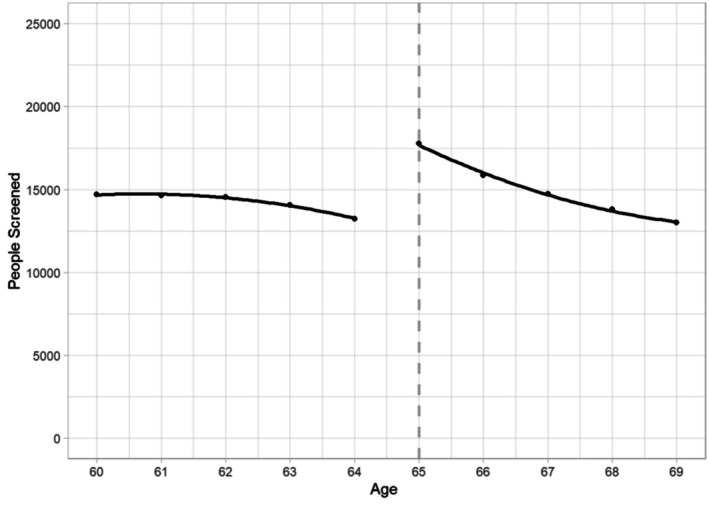
Number of people screened by age (overall). Figure shows the number of first‐time screens by age and the discontinuity in screening at age 65.

In the unadjusted data, there is a visible jump at age 65 in the number of initial screens (Figure 1). Adjusted estimates of the increase at age 65, based on the RDD model, are in Table 2. An additional 5450 people initiated screening at age 65 each year (*p* < 0.001), or a 41% increase relative to the number screened at age 64. Relative increases at age 65 were higher in rural than non‐rural areas (1231 screens, 52%, *p* < 0.001 and 4241, 39%, *p* < 0.001, respectively). Increases in screening at age 65 were significant for both men and women (2888 additional screens, *p* < 0.001 and 2600 additional screens, *p* < 0.001, respectively), and for people with 40 or more pack‐years of smoking history, the highest tier we measured (3237 additional screens, *p* < 0.001). Unadjusted data for each group are depicted in the supplemental file.

**TABLE 2:** Initiation of lung cancer screening at age 65: A regression discontinuity analysis.

	Age 64	Change at age 65		
		RDD estimate (95% confidence interval)	Change	*p*
Number of first‐time lung cancer screens				
Overall	13,242	5450 (4911, 5990)	41%	< 0.001
By gender and location				
Male	6986	2888 (2710, 3066)	41%	< 0.001
Female	6220	2600 (2208, 2991)	42%	< 0.001
Rural	2363	1231 (1138, 1323)	52%	< 0.001
Non‐rural	10,881	4241 (3781, 4701)	39%	< 0.001
By lung cancer risk factors				
Eligible for screening based on USPSTF criteria	11,777	4853 (4431, 5274)	41%	< 0.001
USPSTF eligible, with smoking history of 30–39 pack‐years	3275	1534 (1424, 1643)	47%	< 0.001
USPSTF eligible, with smoking history of 40 or more pack‐years	7452	3237 (2958, 3516)	43%	< 0.001
Currently smoke	7078	3162 (2838, 3486)	45%	< 0.001
Risk mix of screened individuals				
Lung cancer detected after screening	0.56%	0.07 percentage points (−0.08, 0.22)	11%	0.389

*Note:* RDD is regression discontinuity design. USPSTF is the United States Preventive Services Task Force. Overall and stratified results from negative binomial regression discontinuity models controlling for age (centered at age 65) with a quadratic polynomial specification and triangular kernel weights. RDD estimates are reported as marginal effects. The percentage (%) change reported compares the change in first lung cancer screens with low‐dose computed tomography in relation to screens received at age 64. For lung cancer detection (last row), RDD results are from logistic regression models using the same specification and kernel weights. Data from the American College of Radiology's Lung Cancer Screening Registry (2015–2020).

There was no evidence that this increase in screening at age 65 was accompanied by changes in targeting of screening to high‐risk people. There was no statistically significant change at age 65 in the proportion of screened individuals with lung cancer detected (0.56% at age 64% and 0.63% at 65, *p* = 0.39). Findings were qualitatively similar in alternate specifications (supplemental content).

## 4 Discussion

In national registry data, nearly‐universal access to Medicare at age 65 increased the initiation of screening for lung cancer using LDCT. Increases were significant for both men and women, unlike a previous study using a smaller, not‐nationally‐representative sample that found impacts only for men [32]. Relative increases in lung cancer screening were larger in rural areas, where smoking prevalence is higher. Importantly, these additional lung cancer screens at age 65 did not reduce the targeting of screening to people at high lung cancer risk. There was no change in the proportion of screened people who met USPSTF criteria for recommended screening based on their age and smoking history, and no change in the proportion of screened people who had lung cancer detected. These data speak to current debates about how to expand screening while maintaining appropriate targeting. We found that access to Medicare increases appropriate lung cancer screening for people at high risk without excessively increasing screening for people at low risk [6, 34].

The lung cancer screening registry data have several key advantages. First, the data are based on administrative records and are therefore less subject to recall bias than survey data, particularly regarding screening modality. Second, the data are relatively complete, including every lung cancer screen with LDCT in facilities that accept Medicare payments for lung cancer screening nationwide. Finally, the data include information on cancer detection, permitting a novel analysis of whether screening is well targeted.

However, our findings also highlight potential problems with registry data. The primary purpose of the registry was to document compliance with the Medicare coverage criteria, potentially incentivizing facilities to overreport the use of shared decision‐making, required for Medicare payment [39]. Our estimated shared decision‐making rates differ from those measured in an earlier study using claims data, which found that in 2015–2016 only 9% of screened patients participated in a shared decision‐making visit within 3 months of the lung cancer screening [30]. Future research should examine discrepancies between facilities' processes for reporting information to the registry, billing Medicare for shared decision‐making, and the content of shared decision‐making conversations, the last of which some studies have found to be highly variable and lacking [40].

Our finding that many high‐risk adults might delay LDCT screening until age 65 is important for public health because screening reduces lung cancer mortality. Several mechanisms are plausible. First, the number of uninsured individuals aged 64 is high, approximately 10% of 6.1 million in 2023 [41, 42], and those who use tobacco are more likely to be uninsured [43]. This group may benefit the most from gaining Medicare. Second, many people younger than 65 are covered by commercial health plans, a growing proportion of which have deductibles [44]. Individuals enrolled in plans with deductibles are less likely to use lung cancer screening [20, 21]. Concerns about the downstream costs of additional testing (e.g., a biopsy) or treatment could contribute to delays in care until gaining Medicare. Third, Medicare encourages screening via wellness visits designed to increase preventive care, and prior studies have shown uptake in cancer screening associated with the wellness visits [27, 28, 29, 31]. Finally, Medicare represents a large share of revenues for rural hospitals [45], consistent with our finding that proportional increases in screening were larger in rural areas [46]. Understanding the mechanisms by which Medicare increases lung cancer screening rates may help further increase screening in rural areas among people under 65. Given our research design, stigma and changes in patient preferences are unlikely mechanisms explaining our findings.

The validity of the regression discontinuity analysis relies on the assumption that no other change at age 65 caused more people to receive screening. Because the registry data only include screened individuals, they are not well‐suited to test this assumption. However, data from other studies provide some reassurance. Prior work using population‐representative survey data found no change in lung cancer risk (based on pack‐years of smoking history) at age 65 [32]. Furthermore, other studies have shown that education, employment status, and other relevant characteristics do not exhibit a discontinuity at age 65 [32]. To address the concern that underlying cancer risk could change at age 65, cancer mortality shows a discontinuity at age 65 in the United States but not in Canada, where residents experience no change in insurance at age 65 [22].

This study has limitations. First, our research design does not allow us to uncover the mechanisms that drive the increase in screens due to data limitations, and the registry data lack reliable or detailed insurance information. Second, we could not stratify data by race and ethnicity or by level of education due to missingness in the data. Third, the current study design identified changes in lung cancer screening associated with the *onset* of Medicare insurance coverage at age 65. The results may not generalize to other ages.

In summary, nearly universal Medicare coverage increases the initiation of lung cancer screening among people at high lung cancer risk. This finding suggests the need to improve the utilization of lung cancer screening for people younger than 65 who are at high risk but who may delay screening until gaining Medicare coverage.

## Conflicts of Interest

Studts has provided consulting to Genentech and Johnson & Johnson regarding efforts to facilitate implementation of lung cancer screening. Other authors have no conflict to declare.

## Supporting information


**Appendix A1.** Supporting Information.

## Data Availability

The data that support the findings of this study are available from the American College of Radiology. Restrictions apply to the availability of these data, which were accessed by the American College of Radiology analysts. Information on data is available at https://www.acr.org/Practice‐Management‐Quality‐Informatics/Registries/Lung‐Cancer‐Screening‐Registry.
