# Supplementary material for: Delaying Screening Until Covered? Changes in Lung Cancer Screening at the Age of Nearly‐Universal Medicare Insurance
Source: Health Serv Res. 2025 May 8;61(2):e14638. doi: 10.1111/1475-6773.14638 (PMC12967913; doi:10.1111/1475-6773.14638)
Supplement: Supplementary file 1 — Appendix A1. Supporting Information. [file HESR-61-0-s001.docx]

**Supplemental Content**

**Figure A1. Number of People Initiating Screening by Age and Gender**

**A. Men**


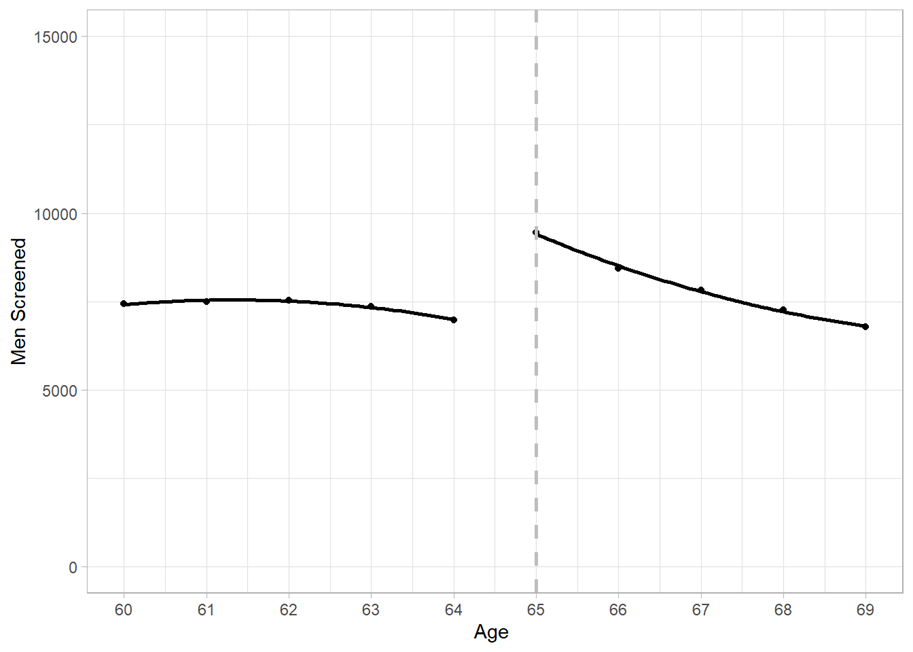


**B. Women**


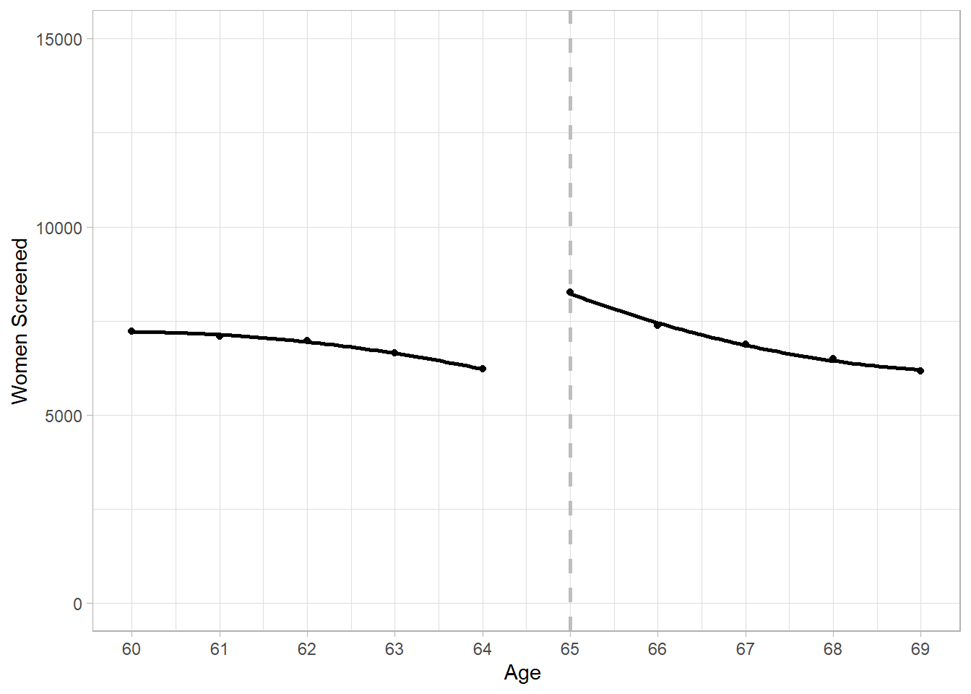


**Figure A2. Number of People Initiating Screening by Age and Location**

**A. Rural Areas**

**
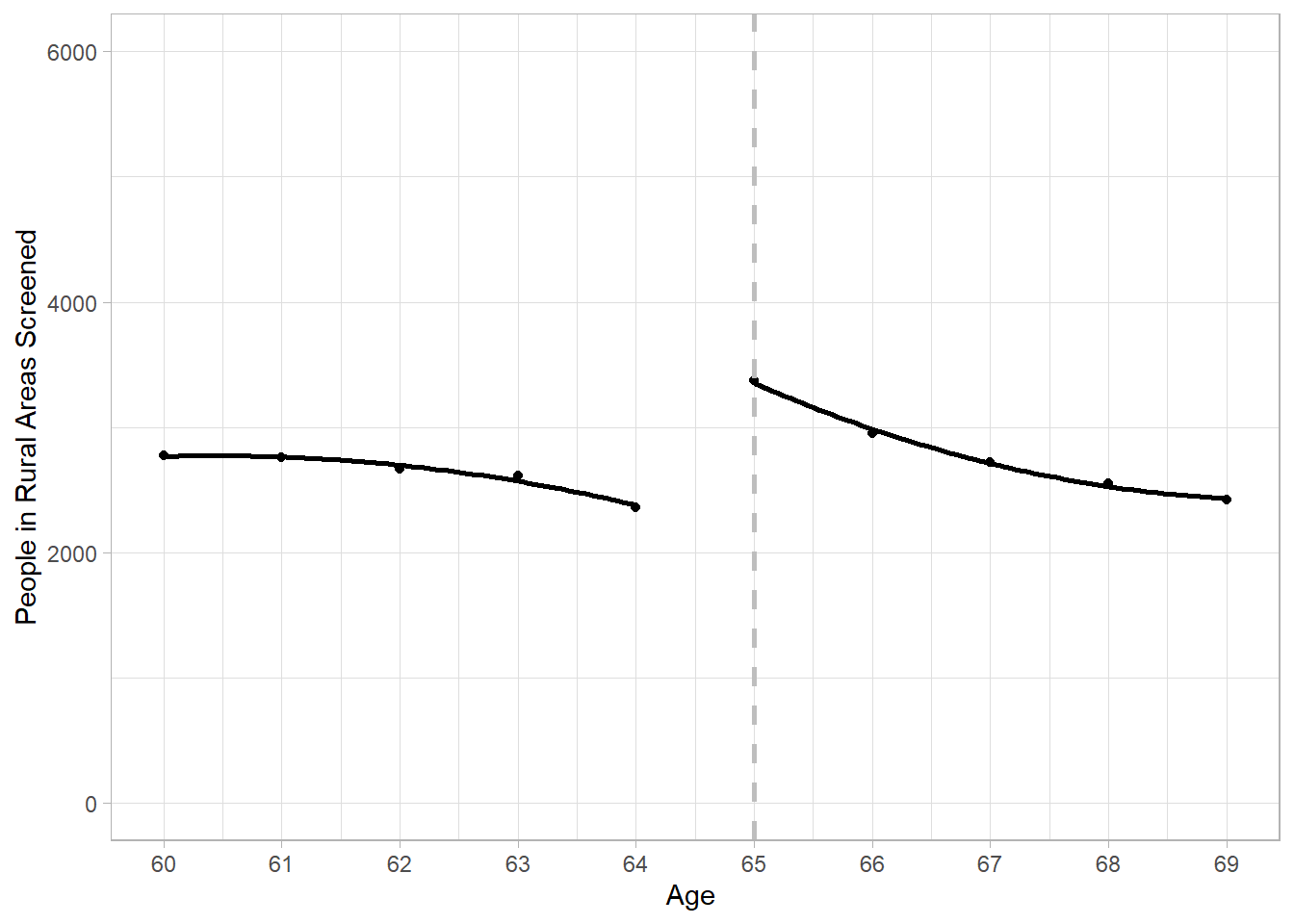
**

**B. Non-Rural Areas**


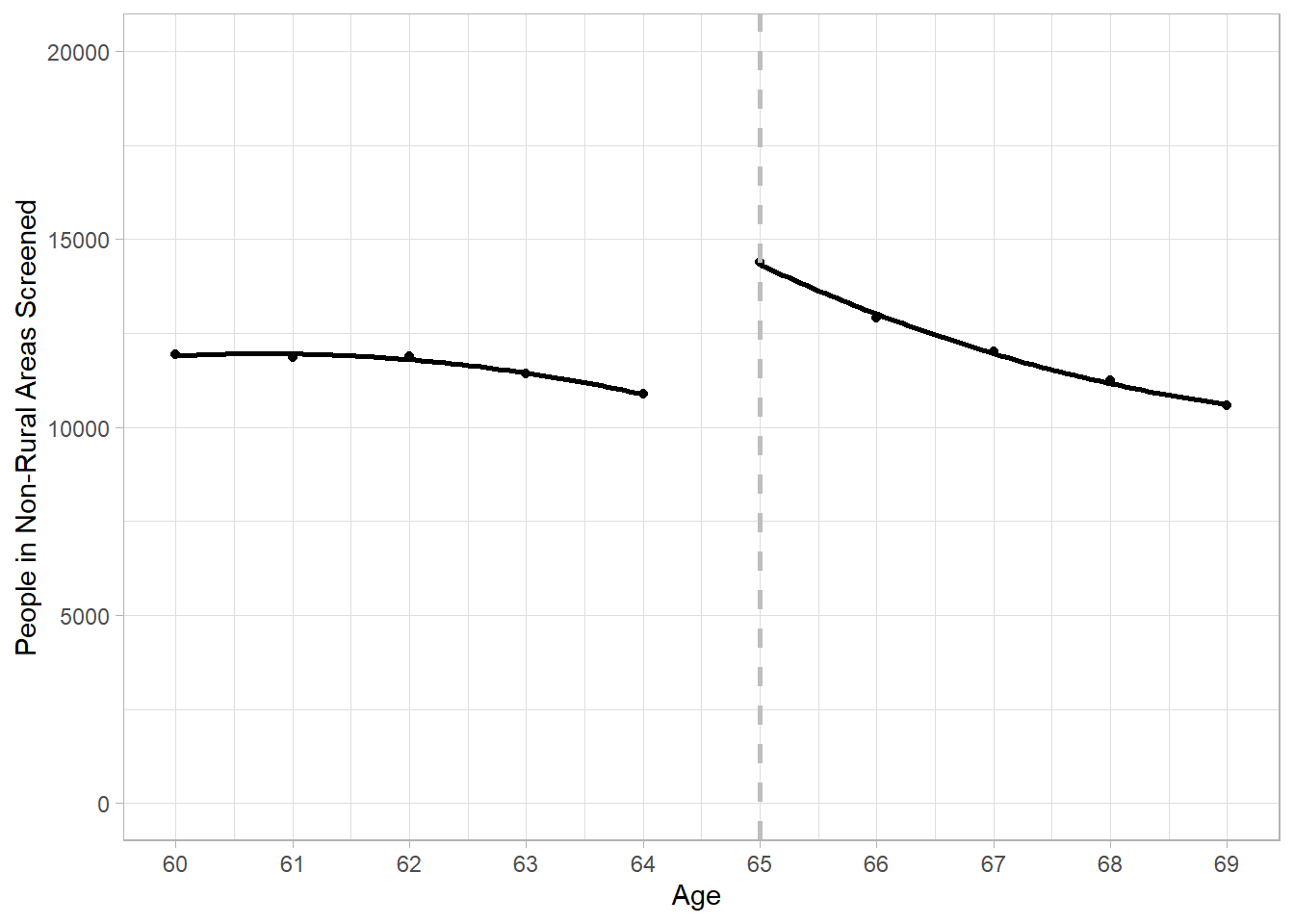


**Figure A3. Number of People Initiating Screening by Age and Ex Ante Measures of Lung Cancer Risk**

**A. Eligible for Screening Based on USPSTF Criteria and Over 40 Pack-Years of Smoking History**


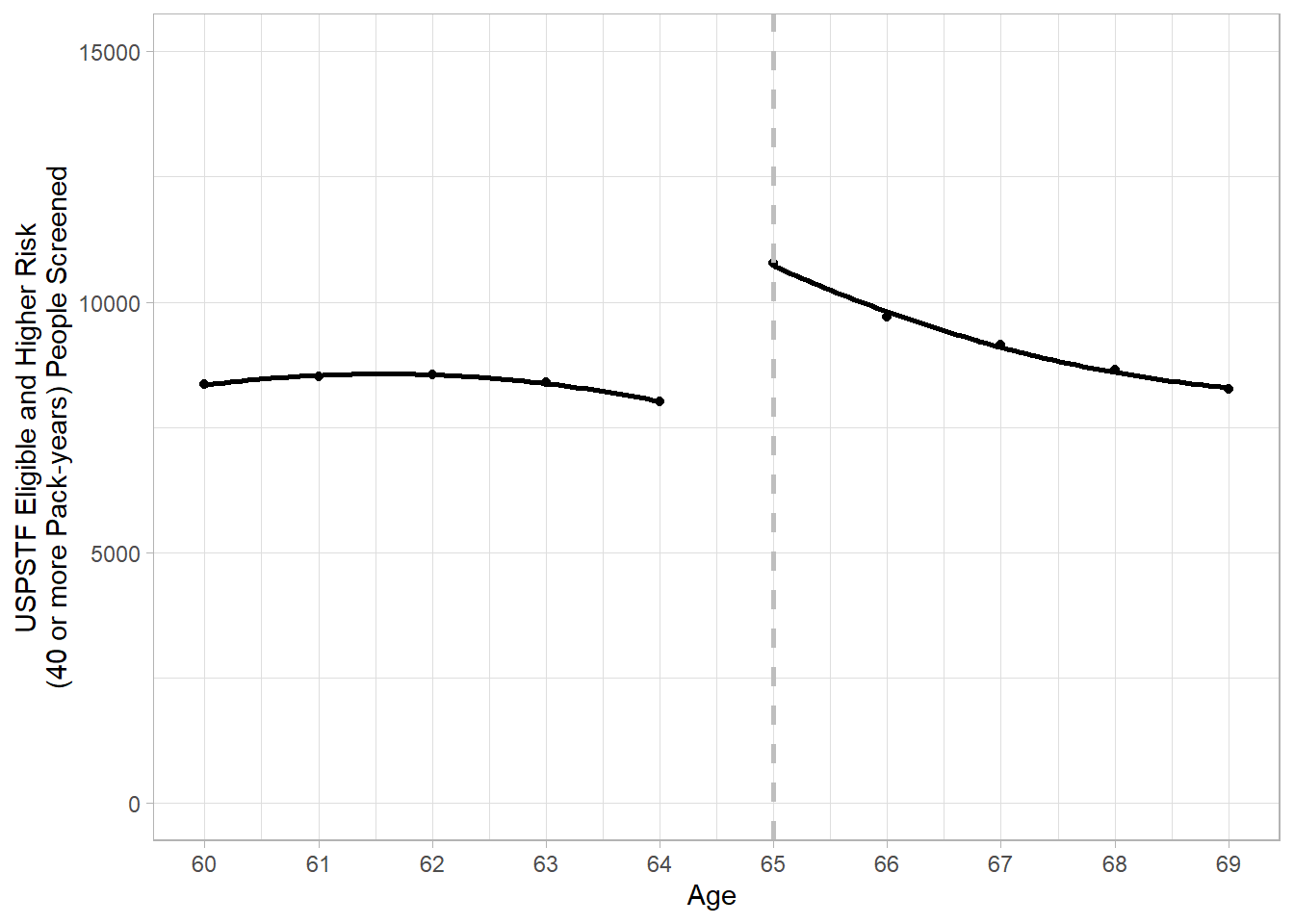


**B. Eligible for Screening Based on USPSTF Criteria and Have 30-39 Pack-Years of Smoking History**


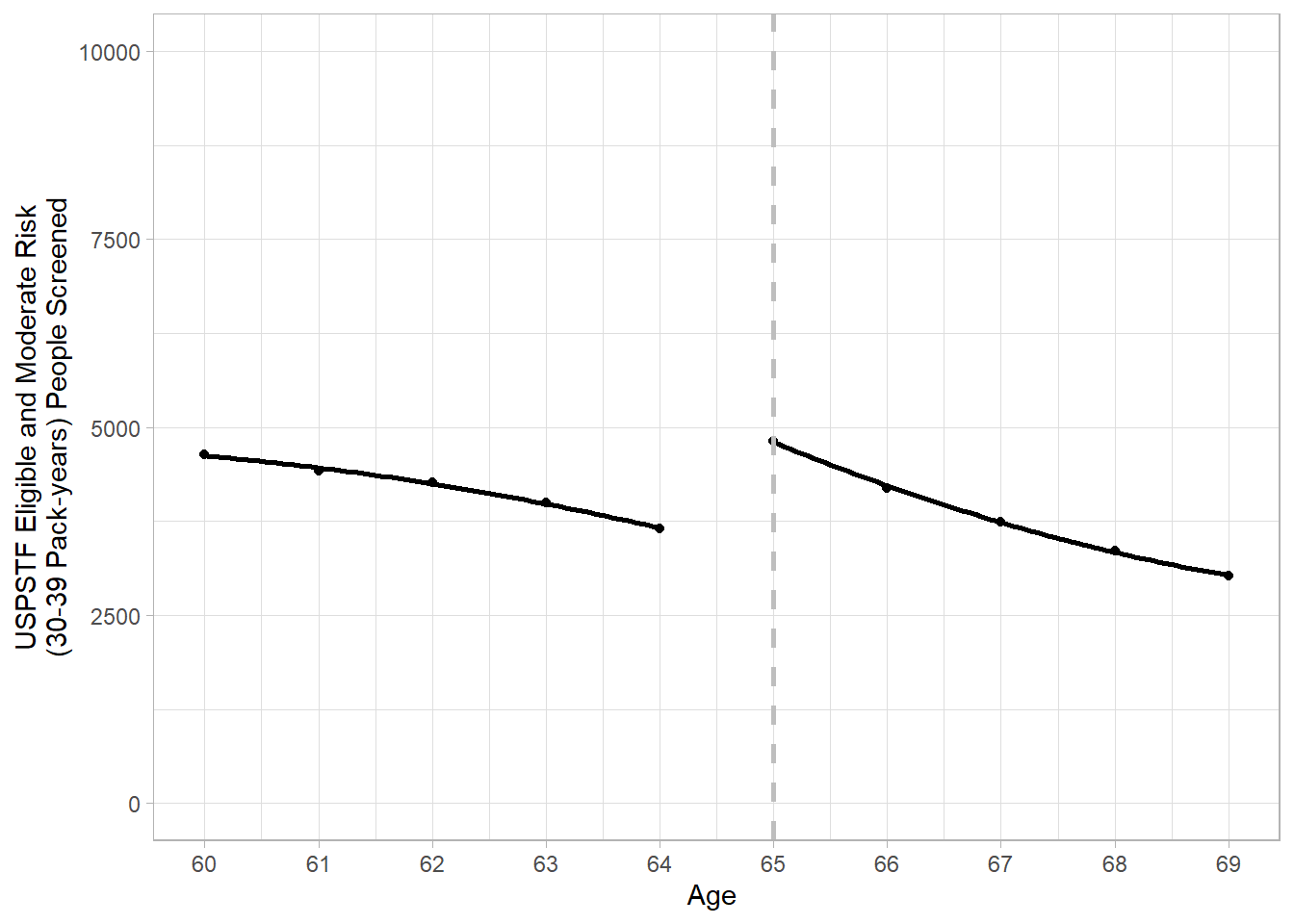


**C. Currently Smoke**


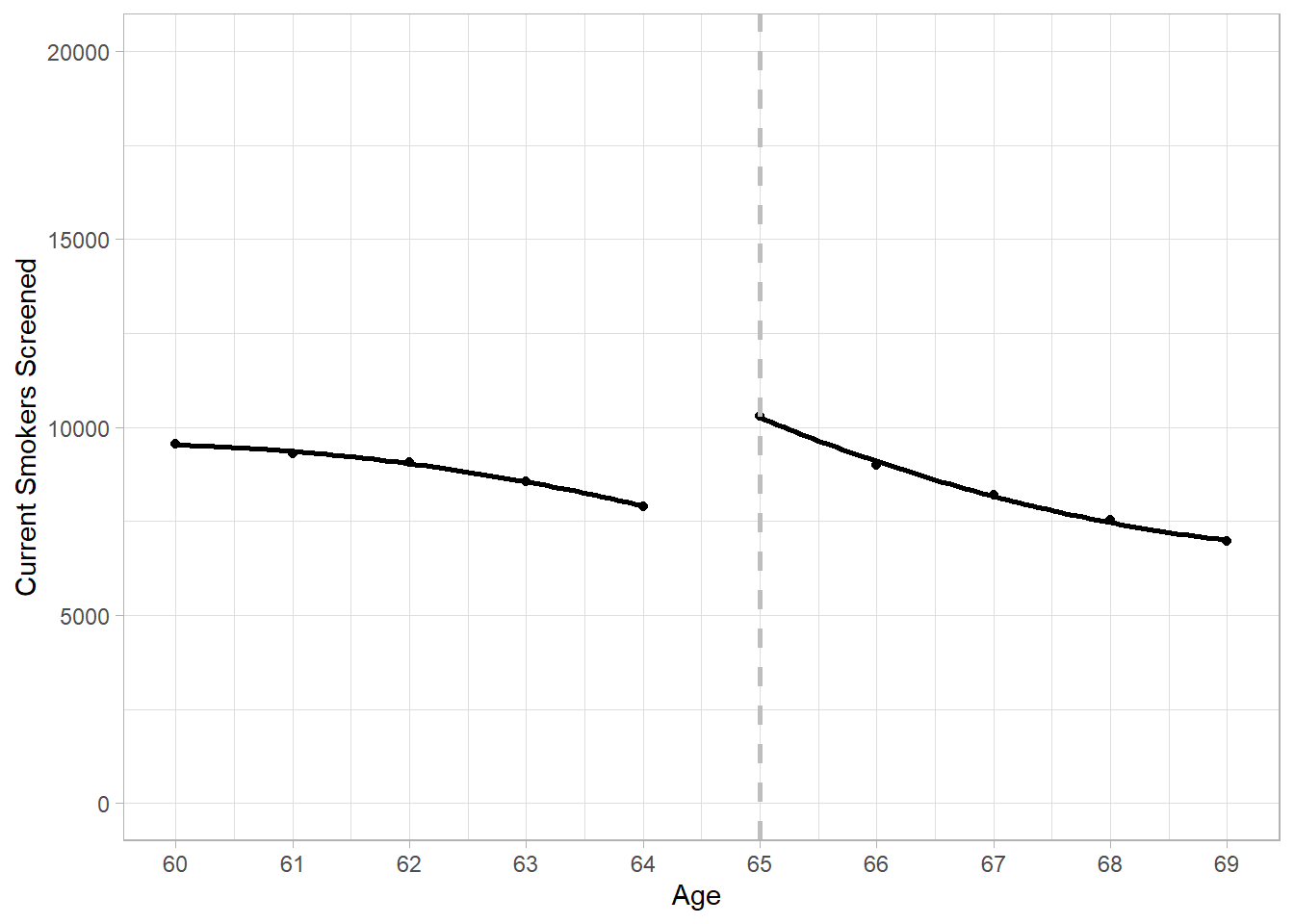


**Figure A4: Proportion of Screened People with Lung Cancer Detected**


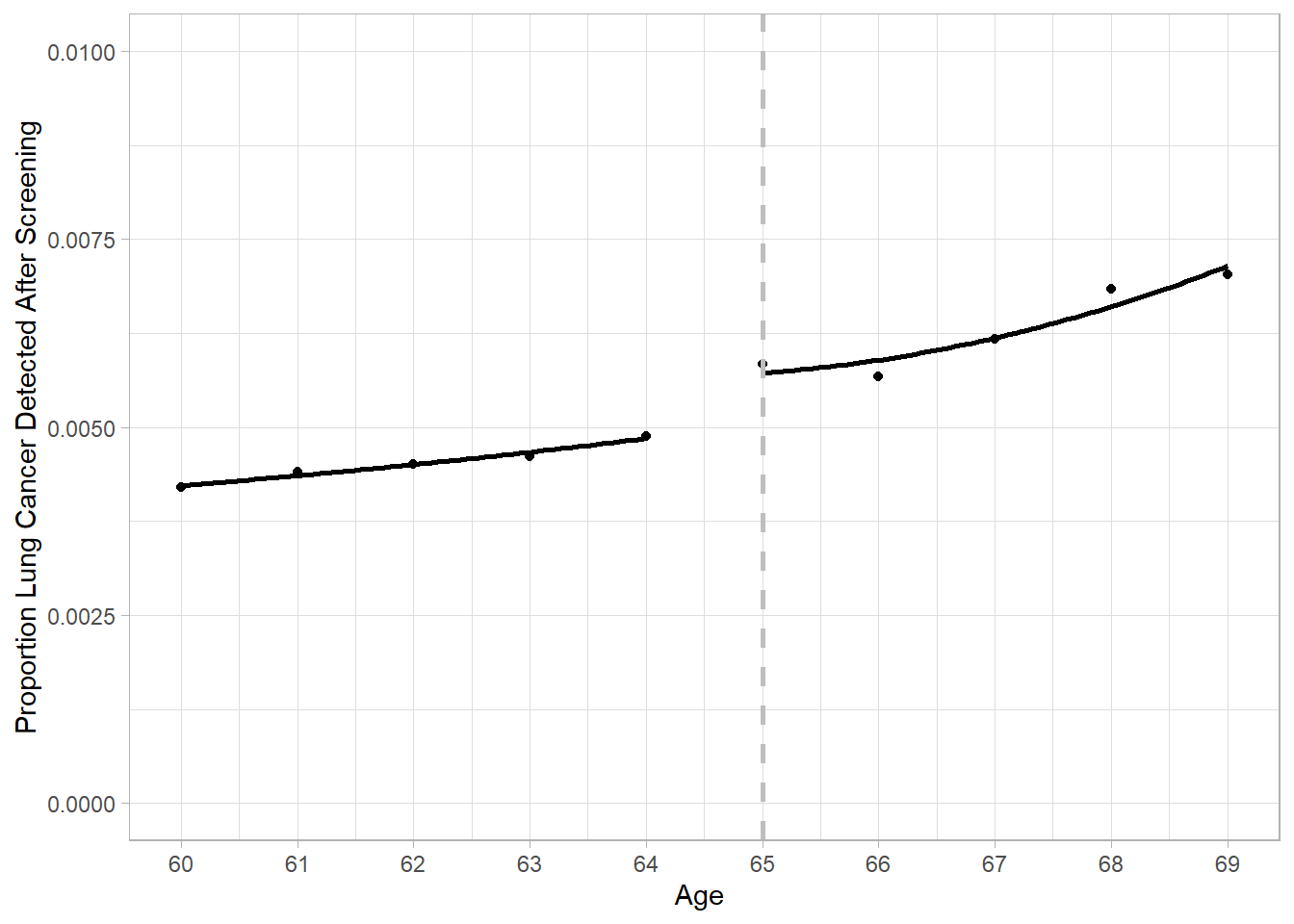


**Table A1: Sensitivity Analyses**

|  | Main specification | Larger bandwidth (6) | Smaller bandwidth (4) | Linear specification of age | No weights |
| --- | --- | --- | --- | --- | --- |
| Number of first-time lung cancer screens |  |  |  |  |  |
| *Overall* |  |  |  |  |  |
| RDD | 5450.02 | 5370.29 | 5646.89 | 4341.01 | 5242.00 |
| 95% CI | 4910.71, 5989.34 | 4796.61, 5943.10 | 5160.18, 6133.57 | 3616.93, 5065.09 | 4483.01, 6001.00 |
| *P* | <0.001 | <0.001 | <0.001 | <0.001 | <0.001 |
| *Male* |  |  |  |  |  |
| RDD | 2888.10 | 2819.77 | 2998.41 | 2236.19 | 2772.47 |
| 95% CI | 2710.01, 3065.72 | 2580.04, 3059.51 | 2829.76, 3167.10 | 1780.73, 2691.64 | 2471.72, 3073.22 |
| *p* | <0.001 | <0.001 | <0.001 | <0.001 | <0.001 |
| *Female* |  |  |  |  |  |
| RDD | 2599.47 | 2566.39 | 2698.81 | 2097.18 | 2493.15 |
| 95% CI | 2207.60, 2991.34 | 2197.75, 2935.04 | 2280.13, 3117.49 | 1773.55, 2420.81 | 2013.30, 2973.00 |
| *p* | <0.001 | <0.001 | <0.001 | <0.001 | <0.001 |
| *Rural* |  |  |  |  |  |
| RDD | 1230.83 | 1193.24 | 1290.85 | 910.97 | 1177.29 |
| 95% CI | 1137.69, 1323.96 | 1083.10, 1302.48 | 1184.61, 1397.08 | 752.38, 1069.55 | 1055.25, 1299.33 |
| *p* | <0.001 | <0.001 | <0.001 | <0.0001 | <0.0001 |
| *Non-rural* |  |  |  |  |  |
| RDD | 4241.15 | 4202.59 | 4347.57 | 3443.50 | 4112.51 |
| 95% CI | 3781.80, 4700.50 | 3734.31, 4670.90 | 3926.62, 4768.51 | 2846.00, 4040.10 | 3482.95, 4742.08 |
| *p* | <0.001 | <0.001 | <0.001 | <0.001 | <0.001 |
| By lung cancer risk factors |  |  |  |  |  |
| *Eligible for screening based on USPSTF criteria* | |  |  |  |  |
| RDD | 4852.70 | 4785.11 | 5023.21 | 3847.99 | 4668.47 |
| 95% CI | 4431.33, 5274.08 | 4320.35, 5249.87 | 4649.61, 5396.80 | 3196.13, 4499.84 | 4049.59, 5287.36 |
| *p* | <0.001 | <0.001 | <0.001 | <0.001 | <0.001 |
| *USPSTF eligible, with smoking history of 30-39 pack-years* | |  |  |  |  |
| RDD | 1533.59 | 1461.24 | 1574.53 | 1272.97 | 1487.25 |
| 95% CI | 1423.76, 1643.41 | 1333.81, 1588.68 | 1467.13, 1681.93 | 1065.50, 1480.45 | 1325.28, 1649.22 |
| *p* | <0.001 | <0.001 | <0.001 | <0.001 | <0.001 |
| *USPSTF eligible, with smoking history of 40 or more pack-years* | |  |  |  |  |
| RDD | 3236.74 | 3289.25 | 3321.23 | 2509.51 | 3152.31 |
| 95% CI | 2957.55, 3515.92 | 2985.85, 3592.64 | 3082.88, 3559.57 | 2038.57, 2980.44 | 2738.62, 3566.00 |
| *p* | <0.001 | <0.001 | <0.001 | <0.001 | <0.001 |
| *Currently smoke* |  |  |  |  |  |
| RDD | 3162.07 | 3112.39 | 3251.27 | 2476.83 | 3057.79 |
| 95% CI | 2837.91, 3486.23 | 2762.63, 3462.14 | 2960.65, 3541.89 | 2044.16, 2909.49 | 2601.12, 3514.46 |
| *p* | <0.001 | <0.001 | <0.001 | <0.001 | <0.001 |
| Risk mix of screened individuals |  |  |  |  |  |
| *Lung cancer detected after screening* | |  |  |  |  |
| RDD | 0.0006 | 0.0007 | 0.0006 | 0.0007 | 0.0007 |
| 95% CI | -0.0008, 0.0022 | -0.0005, 0.0020 | -0.0014, 0.0026 | -0.0001, 0.0015 | -0.0005, 0.0019 |
| *p* | 0.3894 | 0.2196 | 0.5687 | 0.1070 | 0.2520 |

**Notes**: RDD is regression discontinuity design. USPSTF is the United States Preventive Services Task Force. Results from negative binomial regression discontinuity models controlling for age (centered at age 65) with a quadratic polynomial specification and triangular kernel weights. RDD estimates are reported as marginal effects. Data from the American College of Radiology’s Lung Cancer Screening Registry (2015-2020).
